# *Alloionema similis* n. sp., a genetically divergent sibling species of *A. appendiculatum* Schneider, 1859 (Rhabditida: Alloionematidae) from invasive slugs in California, USA

**DOI:** 10.1007/s11230-016-9668-2

**Published:** 2016-10-14

**Authors:** Oleksandr Holovachov, Sven Boström, Irma Tandingan De Ley, Rory J. Mc Donnell, Salomon Alvarado, Timothy D. Paine, Paul De Ley

**Affiliations:** 1 Department of Zoology, Swedish Museum of Natural History, Stockholm, Sweden; 2 Department of Nematology, University of California, Riverside, CA USA; 3 Department of Crop and Soil Science, Oregon State University, Corvallis, OR 97331 USA; 4 Department of Entomology, University of California, Riverside, CA USA

## Abstract

A new species of *Alloionema* Schneider, 1859, *A. similis* n. sp., and the known species *A. appendiculatum* Schneider, 1859 were isolated from cadavers of invasive slugs in California. Both species are described based on morphology, morphometrics and molecular data. *Alloionema similis* n. sp. is morphologically very similar to *A. appendiculatum* but can be distinguished by a more posterior position of the excretory pore in the Kleinform females and longer tail in the Kleinform males. Substantial differences between the two species are, however, found in both 18S and 28S rDNA sequences. Sequence analysis revealed unambiguous autapomorphies in nucleotide sequence and secondary structure of rRNA genes, separating *A. appendiculatum* and *A. similis* n. sp. Molecular phylogenies were inferred from concatenated secondary-structure based multiple sequence alignments of nearly complete 18S and the D1-D3 domains of the 28S rRNA genes. Phylogenetic analyses placed these two species as sister taxa in a monophyletic clade, separately from *Neoalloionema tricaudatum* Ivanova, Pham Van Luc & Spiridonov, 2016 and *N. indicum* Nermuť, Půža & Mráček, 2016.

## Introduction

*Alloionema* Schneider, 1859 (Rhabditida: Alloionematidae) was erected by Schneider (1859) for *Alloionema appendiculatum* Schneider, 1859, a nematode associated with the black slug *Arion ater* (Linneaus), in Germany. In a later paper, Schneider (1866) described the same species under the name *Leptodera appendiculata* Schneider, 1866 with a more detailed morphological description and some illustrations, apparently he erroneously marked it as a new species. Claus (1868) published an extensive account of its morphology and reproduction as well as the alternation of two different saprophytic generations previously reported by Schneider (1859). These two forms are distinguished mainly by their size and tail shape and Mengert (1953) referred to them as “Großform” and “Kleinform”. Chitwood & McIntosh (1934) described from the gastropod host *Succinea avara* Say a variety, *A. appendiculatum* var. *dubia* Chitwood & McIntosh, 1934, intermediate in size between the two forms. Nermuť et al. (2015) made a re-description of *A. appendiculatum* based on material isolated from the invasive slug *Arion vulgaris* Moquin-Tandon (= *A. lusitanicus* Mabille) collected in the Czech Republic.

The first report of the genus in the United States was *A. appendiculatum* var. *dubia* recovered in 1934 from *Succinea avara* in Piscataway, Maryland (Chitwood & McIntosh, 1934). In 2007, surveys of slug nematode parasites in the USA (Ross et al., 2010) yielded a low nematode recovery (5.4%) with the majority (10 of 14) of species of Rhabditidae Örley, 1880 unidentified. Although found most often (34% of all isolates), *A. appendiculatum* was reported only from the states of Oregon (four sites) and Washington (two sites) but from neither of two sites sampled in California.

The first population of *Alloionema* spp. from California was recovered in 2006 from *Arion rufus* (Linnaeus) collected in Eureka. Specimens belonging to Großform were prepared for morphological and molecular studies but the culture was lost, making it impossible to study Kleinform specimens. A subsequent statewide gastropod survey in 2013 resulted in the recovery of multiple *Alloionema* isolates from *Deroceras reticulatum* (Müller) (four isolates), *Lehmannia valentiana* (Férussac) (three isolates) and *Arion hortensis* species complex (the latter complex comprises *A. hortensis* Férussac, *A. distinctus* Mabille and *A. owenii* Davies) (four isolates) collected in San Mateo. The most recent population was isolated from *Arion rufus* collected in McKinleyville, California during a 2015 survey.

With the exception of one isolate (ITD225), which was lost before it could be subjected to sequencing, all populations from 2013 sampling were found to be genetically identical to each other on the basis of their rRNA genes (nearly full length 18S rRNA gene and partial 5′ section of 28S rRNA gene encompassing D1, D2 and D3 domains), but different from previously described populations of *Alloionema appendiculatum* from Europe (Laznik et al., 2009, 2010; Nermuť et al., 2015; Ross et al., 2010; Spiridonov et al., personal communication) as well as from the populations collected in Eureka in 2006 and in McKinleyville in 2015.

The objectives of this paper were: (i) to describe the two genotypes of *Alloionema* from California, giving additional information on morphology, morphometrics and genetic variability of the genus; (ii) to compare the present material with previously described populations of *A. appendiculatum*; and (iii) to designate a new species for the genetically divergent population of *Alloionema* collected in San Mateo in 2013.

## Materials and methods

*Collection and maintenance of gastropods*

Statewide invasive slug and snail surveys were conducted during 2006, 2007, 2013, 2014 and 2015 in California. Gastropods were collected primarily from nurseries and garden centers by examining the area under potted plants and taxa were identified using Mc Donnell et al. (2009). Gastropod specimens collected during these surveys yielded a total of 13 strains of *Alloionema* (Table 1). The first population was collected in 2006 from *Arion rufus* in Eureka while the most recent sample was also recovered from *A. rufus* collected in McKinleyville in 2015. In addition to *A. rufus*, specimens of *Alloionema* were recovered from *A. hortensis* agg., *D. reticulatum* and *L. valentiana* in California. Slugs and snails were reared on organic carrots in plastic containers (26.5 × 15.5 × 6.5 cm) lined with damp paper towel, and following death of the animals, the cadavers were placed on 1% plain agar. Nematodes that emerged were isolated, subcultured, and subsequently maintained on fresh plain agar and nutrient agar (Tandingan De Ley et al., 2014). Our attempts to obtain a Großform by inoculating slugs with Kleinform specimens (isolates 175 and 295) failed; nematodes continued to propagate, the host died, but no Großform could be found in our cultures after the death of the host.

**Table 1:** *Alloionema* isolates, their slug hosts, locality data and sequence availability

Code	Slug host	Location	Collected	18S rRNA	28S rRNA
400/402	*Arion rufus* (Linnaeus)	Eureka, CA	26.vi.2006	**+**	**+**
ITD041	*Deroceras reticulatum* (Müller)	San Mateo, CA	27.i.2013	**+**	**+**
ITD175	*Arion hortensis* agg.	San Mateo, CA	27.i.2013	**–**	**+**
ITD176	*Arion hortensis* agg.	San Mateo, CA	27.i.2013	**–**	**+**
ITD197	*Lehmannia valentiana* (Férussac)	San Mateo, CA	27.i.2013	**+**	**+**
ITD216	*Deroceras reticulatum* (Müller)	San Mateo, CA	27.i.2013	**+**	**+**
ITD219	*Arion hortensis* agg	San Mateo, CA	27.i.2013	**–**	**+**
ITD220	*Arion hortensis* agg.	San Mateo, CA	27.i.2013	**+**	**+**
ITD225	*Deroceras reticulatum* (Müller)	San Mateo, CA	27.i.2013	**–**	**–**
ITD226	*Deroceras reticulatum* (Müller)	San Mateo, CA	27.i.2013	**–**	**+**
ITD294	*Lehmannia valentiana* (Férussac)	San Mateo, CA	27.i.2013	**–**	**+**
ITD295	*Lehmannia valentiana* (Férussac)	San Mateo, CA	27.i.2013	**–**	**+**
ITD792	*Arion rufus* (Linnaeus)	McKinleyville, CA	18.v.2015	**–**	**+**

*Light and scanning electron microscopy*

Nematodes were picked from dead slugs and culture plates, relaxed by gentle heat and fixed in cold 4% formaldehyde solution. For light microscopy (LM), specimens were transferred to pure glycerine by a slow evaporation method and mounted on permanent slides in glycerine with paraffin wax as support for the coverslip. Specimens used in this study are deposited in the general invertebrate collection (slides # SMNH-153525–SMNH-153536) of the Department of Zoology, Swedish Museum of Natural History, Stockholm, Sweden. For scanning electron microscopy (SEM), specimens from the isolate ITD176 were post-fixed in 1% osmium tetroxide (OsO_4_) and transferred to pure acetone through an acetone/distilled water series. Specimens were critical point dried in liquid CO_2_, mounted on stubs, gold-plated under vacuum to a thickness of 200 Å in an Agar High Resolution Sputter Coater Model 20, and examined in a Hitachi S-4300 SEM at an accelerating voltage of 5 kV. All measurements in the descriptions and tables are in micrometres unless otherwise indicated.

*Molecular procedures*

DNA extraction and amplification were performed as described in Tandingan De Ley et al. (2007) for the 5′ section of the 28S (covering either D2-D3 or D1-D2-D3 expansion segments) and the 18S rRNA genes (Tandingan De Ley et al., 2002). Genomic template DNA (2–3 µl) was used in a 25 µl PCR reaction using Illustra PuReTaq Ready-To-Go™ PCR beads (GE Healthcare, 800 Centennial Ave., P.O. Box 1327, Piscataway, NJ, USA) under the same PCR conditions, and using the same amplification and sequencing primers previously described (Blaxter et al., 1998; Tandingan De Ley et al., 2002). Contiguous sequences were assembled and compared with published sequences in the GenBank database using CodonCode Aligner (CodonCode Corp., 58 Beech Street, Dedham, MA, USA).

*Sequence alignment*

The secondary structure alignment was created based on existing secondary structure models of nearly complete 18S and partial 28S rRNA genes as described in Holovachov et al. (2015). New rRNA sequences (Table 2) were added to existing secondary structure-based alignments and aligned to maximize apparent positional homology of nucleotides. Secondary structure annotation was manually added to non-annotated sequences using 4SALE (Seibel et al., 2006); complementarity of base pairings in stem regions was manually verified for all sites.

**Table 2:** GenBank accession numbers for sequences of nematode species used in the phylogenetic analysis

Species	18S rRNA	Partial 28S rRNA
*Rhabditophanes* sp. KR3021	AF202151	KU180691
*Rhabditophanes* sp. 57H6	JX674037	JX674035
*Rhabditophanes* sp. 57H7	JX674037	JX674036
*Strongyloides stercoralis* Bavay, 1876	AF279916	DQ145661
*Strongyloides procyonis* Little, 1966	AB205054	AB205054
*Alloionema appendiculatum* Schneider, 1859 Al	KJ851579	KJ851578
*Alloionema appendiculatum* Schneider, 1859 PE	KP204844	KP204846
*Neoalloionema indicum* Nermuť, Půža & Mráček, 2016	KP204845	KP204847
*Alloionema* strain 400/402	KX185607	KX185601
*Alloionema* strain ITD041	KX185603	KX185591
*Alloionema* strain ITD197	KX185604	KX185594
*Alloionema* strain ITD216	KX185605	KX185595
*Alloionema* strain ITD220	KX185606	KX185597
*Neoalloionema tricaudatum* Ivanova, Pham Van Luc & Spiridonov, 2016	KR817916	KR817917

*Sequence comparison*

Secondary structure-based alignments of all recent and published 18S and 28S rDNA sequences of *Alloionema* and *Neoalloionema* Ivanova, Pham Van Luc & Spiridonov, 2016 (Table 3) were visually compared in SeaView (Gouy et al., 2010). For comparative analysis, consensus sequences were created for *A. appendiculatum* and *A. similis* n. sp. Common sites were excluded from all sequences of *A. similis* n. sp., *Neoalloionema indicum* and *N. tricaudatum*, while variable sites were retained.

**Table 3:** Sequences used for comparison of primary rRNA structure (Figs. 6 and 7)

Species	18S rRNA	partial 28S rRNA	Host or origin	Reference
*A. appendiculatum* consensus:				
*A. appendiculatum*	EU573707	–	*Arion lusitanicus*	Ross et al. (2010)
*A. appendiculatum*	FJ516751	–	unknown	Spiridonov et al. (unpublished data)
*A. appendiculatum* “Slovenia”	FJ665982	–	*A. lusitanicus*	Laznik et al. (2009, 2010)
*A. appendiculatum* Al	KJ851579	KJ851578	*Arion vulgaris* ^a^	Nermuť et al. (2015)
*A. appendiculatum* PE	KP204844	KP204846	unknown	Nermuť et al. (2015)
*A. appendiculatum* 400/402	KX185607	KX185601	*Arion rufus*	This study
*A. appendiculatum* ITD792	–	KX185602	*A. rufus*	This study
*A. similis* consensus:				
*A. similis* ITD041	KX185603	KX185591	*Deroceras reticulatum*	This study
*A. similis* ITD175	–	KX185592	*Arion hortensis* agg.	This study
*A. similis* ITD176	–	KX185593	*A. hortensis* agg.	This study
*A. similis* ITD197	KX185604	KX185594	*Lehmannia valentiana*	This study
*A. similis* ITD216	KX185605	KX185595	*D. reticulatum*	This study
*A. similis* ITD219	–	KX185596	*A. hortensis* agg.	This study
*A. similis* ITD220	KX185606	KX185597	*A. hortensis* agg.	This study
*A. similis* ITD226	–	KX185598	*D. reticulatum*	This study
*A. similis* ITD294	–	KX185599	*L. valentiana*	This study
*A. similis* ITD295	–	KX185600	*L. valentiana*	This study
*Neoalloionema indicum*	KP204845	KP204847	Félix Lab	Nermuť et al. (2015)
*Neoalloionema tricaudatum*	KR817916	KR817917	*Cyclophorus* sp.	Ivanova et al. (2016)

^a^ *Arion vulgaris* (= *Arion lusitanicus*)

*Visualization of rRNA secondary structure*

Secondary structures of selected domains of both 18S rRNA and 28S rRNA were visualized with the aid of VARNA (Darty et al., 2009), saved as vector graphics and converted into raster graphic format for publication.

*Phylogenetic analysis*

The concatenated alignment was analyzed with Bayesian phylogenetic inference using the *mcmcphase* program in the PHASE 2.0 package (Gowri-Shankar & Jow, 2006). The entire concatenated alignment was partitioned into 18S rDNA and 28S rDNA partitions. Furthermore, each partition was divided into secondary partitions of “stems” (paired sites) and “loops” (non-paired sites) to account for the potential phylogenetic importance of compensatory substitutions. The REV nucleotide substitution model (Tavaré, 1986) was used for non-paired sites, whereas RNA7A (Higgs, 2000) nucleotide substitution model was used for paired sites. Model parameters were estimated independently for all sub-partitions (non-paired and paired sites of 18S rRNA gene and non-paired and paired sites of partial 28S rRNA gene). Chains were allowed to burn in for 500,000 generations, followed by 5 million generations (total 5.5 million generations) during which tree topologies, branch length and model parameters were sampled every 200 generations. The tree was rooted using *Rhabditophanes* sp. KR3021.

**Family Alloionematidae Chitwood & McIntosh, 1934**

**Genus** ***Alloionema*** **Schneider, 1859**

***Alloionema appendiculatum*** **Schneider, 1859**

### Description of Großform from *Arion rufus* (Figs. 1A, C, 2A–C)

*Host*: *Arion rufus* (Linnaeus).

**Fig. 1:**
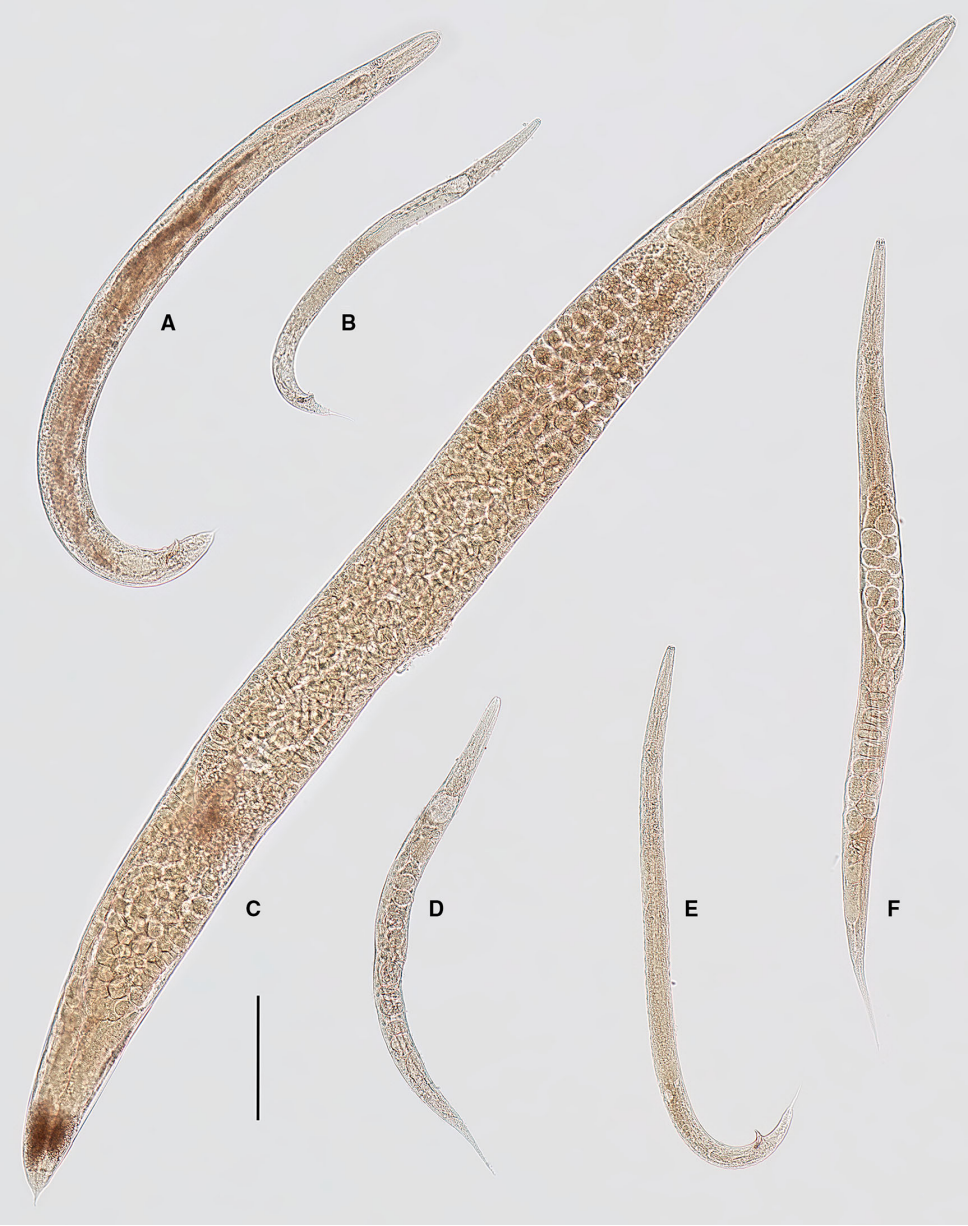
Light microscopy photomicrographs of *Alloionema appendiculatum* Schneider, 1859 and *A. similis* n. sp. Entire specimens. A, *A. appendiculatum*, Großform, male; B, *A. appendiculatum,* Kleinform, male; C, *A. appendiculatum*, Großform, female; D, *A. appendiculatum*, Kleinform, female; E, *A. similis* n. sp., Kleinform, male; F, *A. similis* n. sp., Kleinform, female. *Scale-bar*: 200 µm

**Fig. 2:**
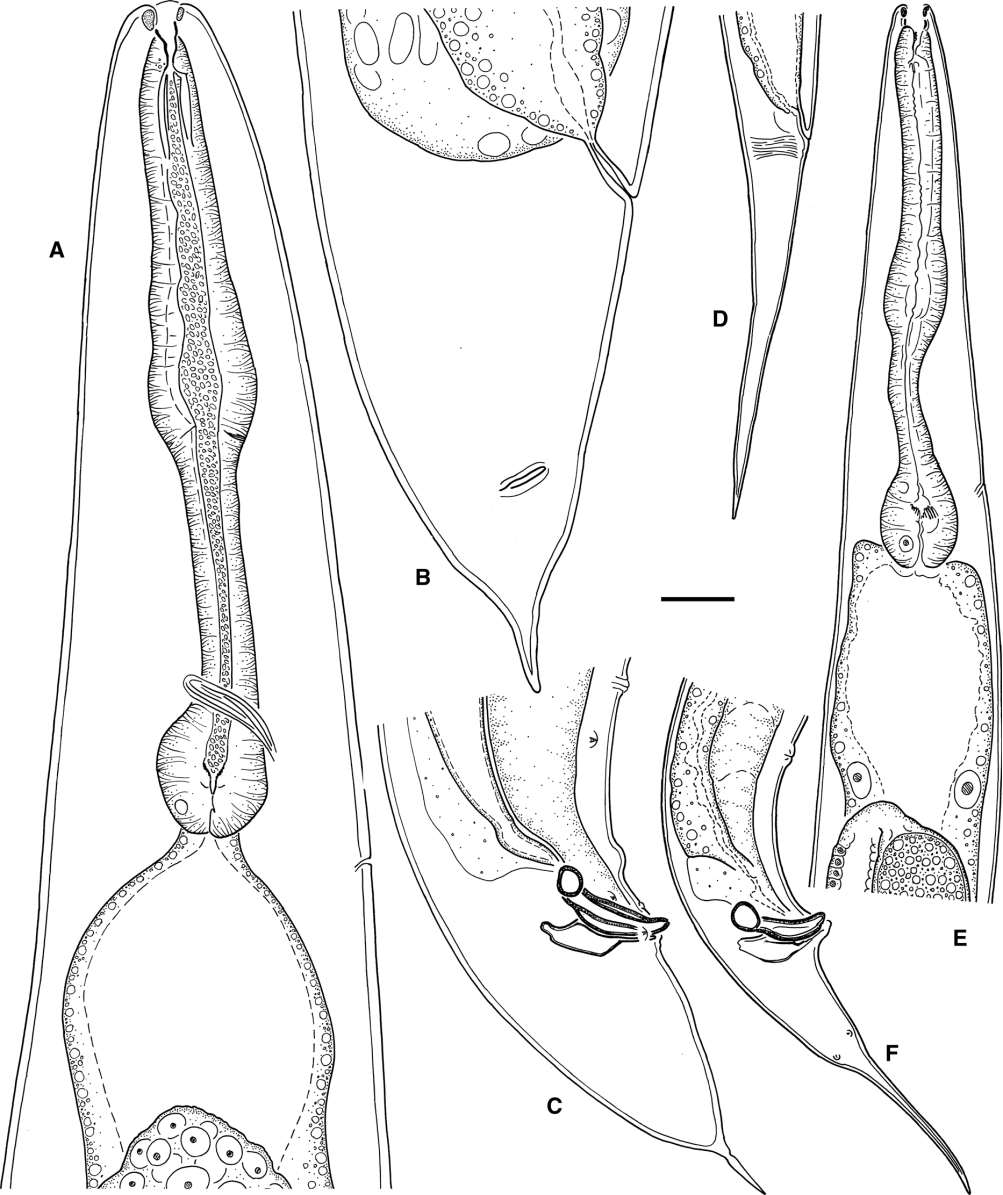
Line drawings of *Alloionema appendiculatum* Schneider, 1859. Großform (A–C) and Kleinform (D–F) generations. A, E, Female, pharyngeal region; B, D, Female, tail; C, F, Male, tail. *Scale-bar*: 20 µm

*Locality*: Potted plant in a garden center in Eureka, California, on 26.vi.2006 (GPS coordinates: 40°46′06′′N, 124°11′33′′W).

*Voucher material*: Two females and four males on slides # SMNH-153527–SMNH-153528 deposited in the general invertebrate collection of the Department of Zoology, Swedish Museum of Natural History, Stockholm, Sweden.

*General* [Based on six specimens; see measurements in Table 4.] Body 2.0–2.4 mm long in females and 1.3-1.5 mm long in males. When killed by heat, females slightly arcuate ventrad and males more strongly arcuate ventrad, especially in posterior end. Cuticle finely annulated, annules less prominent in LM, in anterior body region 2.2–2.4 wide and 1.6–1.7 wide in females and males, respectively. Lateral field not seen in LM or SEM. Lip region rounded, continuous with body contour. Anterior end gradually tapering. Six rounded lips grouped in 3 pairs, 1 dorsal and 2 ventrolateral, carrying 6 inner labial, 6 outer labial and 4 cephalic papilliform sensilla and 2 small oval amphids. Stoma less than one lip region diameter long. Cheilostom broad, with thick rounded rhabdia; gymnostom short; stegostom funnel-shaped, with strongly sclerotised lining. Pharynx muscular; corpus cylindrical, 1.5–2.1 times as long as isthmus, widening posteriorly to a non-valvular metacorpus; isthmus narrower, demarcated by a break in muscular tissue; basal bulb oval, with weakly developed valves. Nerve-ring surrounding isthmus. Excretory pore somewhat more posterior, opening in posterior part of isthmus, at isthmus-bulb junction or at terminal bulb. Deirids not observed.

**Table 4:** Morphometric comparison of Großform generation of *Alloionema* species

Species	*Alloionema appendiculatum* Schneider, 1859						*Alloionema appendiculatum* var. *dubia*	
Reference	This study		Mengert (1953)		Nermuť et al. (2015)		Chitwood & McIntosh (1934)	
Sex	♀♀	♂♂	♀♀	♂♂	♀♀	♂♂	♀♀	♂♂
n	2	4	?	?	20	20	?	?
L	2,036–2,401	1,460 ± 93 (1,304–1,548)	2,264–2,675	1,176–1,617	1,956 ± 290 (1,555–2,525)	1,483 ± 199 (1,111–1,919)	1,400–1,650	954–1,000
a	12.6–14.0	15.6 ± 0.2 (15.4–15.9)	12–15	9–13	14.1 ± 3.3 (8.6–21.0)	15.7 ± 1.9 (13.1–20.0)	–	–
b	10.3–11.4	8.4 ± 0.8 (7.1–9.1)	10–11	7	9.6 ± 1.8 (7.0–14.0)	7.6 ± 1.1 (5.3–10.8)	–	–
c	16.9–17.8	16.9–21.4 (n = 2)	14	13–18	12.8 ± 2.2 (8.8–17.3)	19.6 ± 3.4 (12.9–25.8)	–	–
c′	1.6–1.7	1.5–1.6 (n = 2)	1.6^a^	1.4^a^	1.9 ± 0.3 (1.4–2.4)	2.4 ± 0.4 (1.7–3.4)	–	2.7^a^
V or T (%)	53–54	82.5 ± 2.1 (80–85)	48–57	–	56.2 ± 3.0 (50.1–60.6)	–	53–55^b^	–
Max. body diameter	145–191	94 ± 6.9 (82–100)	147–176	88–132	143 ± 23.8 (101–202)	95 ± 12.5 (80–121)	100–110	55–60
Lip region diameter	25	21.7 ± 1.2 (20–23)	20^a^	–	16.2 ± 2.6 (11.7–19.6)	12.5 ± 1.6 (11.7–15.6)	–	–
Stoma length	14.5–17.0	15.6 ± 0.9 (14.5–17.0)	8–9	6–8	11.0 ± 1.0 (9.5–12.7)	10.1 ± 1.4 (8.0–12.7)	–	–
Corpus length	105–106	94 ± 6.5 (84–102)	108^a^	–	–	–	91–110	
Isthmus length	54–66	54.5 ± 4.3 (48–60)	65^a^	–	57 ± 4.2 (51–66)	54 ± 6.5 (43–66)	40–47	
Basal bulb length	39	28.5 ± 1.5 (27–31)	39^a^	–	29.5 ± 4.2 (19.6–39.1)	30.5 ± 2.9 (27.4–39.1)	40–47	
Basal bulb diameter	29–36	24.3 ± 0.4 (24–25)	30^a^	–	26.0 ± 4.0 (19.6–31.3)	26.8 ± 2.6 (19.6–31.3)	30	
Corpus/isthmus ratio	1.6–1.9	1.7 ± 0.2 (1.5–2.1)	1.7^a^	–	–	–	–	–
Pharynx length	198–211	177 ± 6.5 (167–184)	212^a^	–	196 ± 21.1 (168–235)	192 ± 14.5 (152–227)	136–200	
Nerve-ring to anterior end	151–187	137 ± 12 (121–151)	–	–	169 ± 19.8 (133–196)	153 ± 15.5 (117–180)	–	–
Excretory pore to anterior end	181–239	155 ± 9.3 (145–165)	–	–	196 ± 26.0 (156–250)	153 ± 18.3 (127–183)	–	–
Lip region to vulva	1,088–1,307	–	–	–	–	–	–	–
Vulva-anus/tail	7.1 (n = 1)	–	–	–	–	–	–	–
Vagina length	24 (n = 1)	–	–	–	–	–	–	–
Anal body diameter (ABD)	72–85	47.3 ± 1.3 (45–48)	93^a^	61^a^	34.2 ± 5.4 (23.9–42.9)	33.1 ± 4.3 (23.9–38.2)	–	–
Rectum length (RL)	66 (n = 1)	–	–	–	–	–	–	–
RL/ABD	0.8 (n = 1)	–	–	–	–	–	–	–
Tail length (TL)	121–135	74–77 (n = 2)	153–184	77–102	154 ± 10.9 (121–176)	76 ± 7.3 (59–86)	100	36–46
Anus to phasmid	79–88	–	–	–	–	–	–	–
Phasmid (% TL)	65	–	–	–	–	–	–	–
Testis length	–	1,201 ± 62 (1,102–1,266)	–	–	–	–	–	–
Testis flexure length	–	80 ± 18 (60–101)	–	–	–	98 ± 22.0 (59–129)	–	–
Spicule length	–	38.3 ± 0.9 (37–39)	–	31–39	–	34.6 ± 2.3 (30.2–39.8)	–	36–46
Gubernaculum length	–	33.3 ± 0.5 (33–34)	–	29–34	–	31.2 ± 2.1 (27.0–36.3)	–	–

Measurements in µm and in the form mean ± standard deviation and (range) or only range

^a^Measured on drawings in the original publication; ^b^ Calculated from data in the original publication

*Abbreviations*: L, body length; a, body length divided by greatest body diameter; b, body length divided by the distance from anterior end to pharynx base; c, body length divided by tail length, c′, tail length divided by body diameter at level of anal or cloacal opening; V, position of the vulva from anterior end expressed as a percentage of total body length; T, length of male reproductive system expressed as a percentage of total body length

*Female*. Reproductive system didelphic, amphidelphic, ovaries reflexed. Oviducts filled with sperm. Gonads filled with oöcytes and hatched juveniles. Vulva a transverse slit, vulval lips not protruding; vagina *c*.1/8 of vulval body diameter (VBD). Tail conoid, tapering rapidly posteriorly to a minutely rounded terminus. Rectum short, less than one time anal body diameter (ABD) long. Phasmids in the shape of large transverse slits located at posterior third of tail length.

*Male.* Similar to female in most respects, except for the sexual characters. Reproductive system monorchic, testis reflexed dorsad anteriorly. Spicules paired, with weakly arcuate shaft and manubria bent sideways. Gubernaculum with robust dorsal apophysis. Genital papillae distributed as follows: a single midventral large pad-like precloacal papilla 74–88 anterior to cloaca; 2 subventral precloacal pairs (at 59–64 and 16–22 anterior to cloaca, respectively); single subventral adcloacal pair; other papillae indistinct, if present. Phasmids are not discernible in our specimens. Tail differently shaped than in female, strongly curved ventrad, conoid with an 18–20 long mucro ending in a pointed terminus.

### Remarks

The present material agrees well with the description of the Großform of *A. appendiculatum* by Mengert (1953) (see Table 4). Our study of the Californian specimens also largely corroborates the results obtained by Nermuť et al. (2015) based on Czech material, but there is one notable difference: larger anal body diameter in females and males from California (72–85 *vs* 23–43 µm and 45–48 *vs* 23–38 µm, respectively).

### Description of Kleinform from *Arion rufus* strain ITD792 (Figs. 1B, D, 2D–F)

*Host*: *Arion rufus* (Linnaeus).

*Locality*: Garden center in McKinleyville town center in Northern California on 18.v.2015 (GPS coordinates: 40°56′16′′N, 124°06′05′′W).

*Voucher material*: Twenty females and seventeen males on slides # SMNH-153529–SMNH-153536 deposited in the general invertebrate collection of the Department of Zoology, Swedish Museum of Natural History, Stockholm, Sweden.

*General* [Based on 22 specimens; see measurements in Table 5.] Body 0.7–0.9 mm long in females and 0.6–0.8 mm long in males. When killed by heat, females almost straight and males slightly arcuate ventrad, more strongly arcuate in the posterior end. Cuticle finely annulated, annules less prominent in LM, *c.*0.5 wide. Lateral field not seen in LM. Anterior end gradually tapering. Lip region rounded, continuous with body contour. Six rounded lips grouped in 3 pairs, 1 dorsal and 2 ventrolateral, carrying 6 inner labial, 6 outer labial and 4 cephalic papilliform sensilla and 2 small oval amphids. Stoma somewhat longer than lip region diameter. Cheilostom broad, with thick rounded rhabdia; gymnostom short; stegostom funnel-shaped, with strongly sclerotised lining and small denticles in its dorsal sector. Pharynx muscular; corpus cylindrical, 1.9–2.8 times as long as isthmus, widening posteriorly to a non-valvular metacorpus; isthmus narrower, demarcated by a break in muscular tissue; basal bulb oval, with strongly developed valves. Nerve-ring surrounding isthmus. Excretory pore opening at middle or posterior part of isthmus or anterior part of basal bulb. Deirids not observed.

**Table 5:** Morphometric comparison of Kleinform generation of *Alloionema* species

Species	*Alloionema similis* n. sp.			*Alloionema appendiculatum* Schneider, 1859					
Reference	This study			Mengert (1953)		Nermuť et al. (2015)		This study	
Sex	♀	♀♀ (incl. holotype)	♂♂	♀♀	♂♂	♀♀	♂♂	♀♀	♂♂
n	holotype	5	6	?	?	20	20	14	8
L	1,309	1,254 ± 39 (1,189–1,309)	995 ± 41 (910–1,029)	922–1,073	561–926	1,276 ± 134 (889–1,454)	932 ± 44 (848–1,010)	851 ± 36 (781–905)	734 ± 54 (651–806)
a	18.7	19.4 ± 1.2 (17.8–20.9)	22.7 ± 1.2 (21.3–25.1)	12–16	12–29	16.8 ± 2.5 (12.0–23.8)	18.4 ± 2.7 (14.0–24.5)	16.6 ± 1.0 (15.0–18.8)	20.4 ± 0.8 (19.1–22.1)
b	6.8	6.7 ± 0.3 (6.2–7.1)	5.8 ± 0.2 (5.4–6.2)	5–6	4–6	7.3 ± 1.1 (5.8–9.5)	5.3 ± 0.3 (4.6–5.8)	5.3 ± 0.4 (4.8–6.0)	5.1 ± 0.3 (4.5–5.7)
c	7.9	8.2 ± 0.5 (7.5–8.9)	9.4 ± 0.2 (9.2–9.9)	7–8	6–10	8.1 ± 0.8 (6.5–9.3)	14.2 ± 1.7 (11.4–17.8)	7.6 ± 0.5 (6.6–8.2)	9.2 ± 0.3 (8.6–9.7)
cv	6.9	6.3 ± 0.6 (5.3–6.9)	3.2 ± 0.1 (3.0–3.4)	5.2^a^	2.8^a^	4.9 ± 0.5 (4.0–6.3)	5.3 ± 0.7 (3.9–6.9)	5.6 ± 0.4 (5.0–6.3)	3.0 ± 0.1 (2.9–3.2)
V or T (%)	53	54 ± 1.1 (53–56)	72 ± 3.6 (65–75)	49–53	–	50.8 ± 4.5 (44.4–59.8)	–	52.7 ± 1.2 (51–56)	74.1 ± 3.3 (70–81)
Max. body diameter	70	65 ± 5.5 (56–71)	44 ± 2.6 (41–47)	59–96	29–45	77 ± 6.5 (59–90)	51 ± 6.3 (40–60)	51.4 ± 2.4 (48–56)	36.0 ± 2.4 (33–40)
Lip region diameter	15.5	15.0 ± 0.4 (14.5–15.5)	12.5 ± 0.5 (12–13)	13^a^	–	14.0 ± 1.2 (11.1–15.9)	12.5 ± 1.2 (11.1–14.3)	11.8 ± 0.5 (11–13)	11.1 ± 0.2 (11.0–11.5)
Stoma length	15.5	15.4 ± 0.2 (15.0–15.5)	14.0 ± 0.7 (13.0–14.5)	8–9	8–9	8.7 ± 1.1 (7.9–11.1)	9.4 ± 0.9 (8.0–11.1)	14.8 ± 0.9 (13.0–15.5)	13.5 ± 0.5 (13–14)
Corpus length	112	111 ± 5 (105–117)	101 ± 3.4 (96–107)	93^a^	–	–	–	96.3 ± 5.0 (87–102)	86.3 ± 2.5 (84–90)
Isthmus length	52	49.4 ± 0.2 (45–52)	45.5 ± 2.4 (42–49)	48^a^	–	39 ± 4.3 (33–48)	40 ± 2.9 (35–47)	39.1 ± 4.4 (31–46)	36.4 ± 2.9 (33–42)
Basal bulb length	28	27.8 ± 0.4 (27–28)	24.5 ± 1.1 (24–27)	33^a^	–	31.8 ± 4.7 (23.9–39.8)	28.6 ± 2.3 (23.9–33.4)	25.8 ± 1.7 (23.0–27.5)	22.8 ± 1.4 (20.5–25.5)
Basal bulb diameter	23	23.8 ± 0.7 (23–25)	20.2 ± 0.9 (19–22)	24^a^	–	26.7 ± 4.7 (20.7–36.6)	24.5 ± 3.1 (15.9–32.0)	21.4 ± 1.0 (20.5–23.0)	18.8 ± 1.3 (17.5–22.0)
Corpus/isthmus ratio	2.2	2.3 ± 0.1 (2.1–2.4)	2.2 ± 0.1 (2.2–2.4)	1.9^a^	–	–	–	2.5 ± 0.3 (1.9–2.8)	2.4 ± 0.1 (2.1–2.5)
Pharynx length	192	188 ± 6.1 (178–194)	171 ± 5.0 (165–180)	174^a^	–	174 ± 18.9 (137–215)	164 ± 8.3 (145–176)	161 ± 7.9 (144–171)	144 ± 6.7 (135–153)
Nerve-ring to anterior end	165	161 ± 5.2 (151–165)	146 ± 3.7 (139–151)	–	–	135 ± 13.5 (118–167)	135 ± 4.8 (129–149)	118 ± 5.9 (102–127)	113 ± 3.7 (110–118)
Excretory pore to anterior end	183	181 ± 6.4 (169–188)	171 ± 4.6 (163–176)	–	–	134 ± 17.4 (117–164)	143 ± 11.9 (117–176)	131 ± 7.5 (127–143)	125 ± 9.0 (114–141) (n = 5)
Lip region to vulva	694	681 ± 18 (657–706)	–	–	–	–	–	449 ± 18 (422–485)	–
Vulva-anus/tail	2.8	2.8 ± 0.2 (2.5–3.0)	–	–	–	–	–	2.6 ± 0.1 (2.3–2.8)	–
Vagina length	25	22.0 ± 2.1 (19–25)	–	–	–	–	–	13.9 ± 1.4 (12.0–15.5)	–
Anal body diameter (ABD)	24	24.6 ± 0.8 (24–26)	33.5 ± 0.5 (33–34)	36^a^	45^a^	32.6 ± 3.1 (27.4–39.1)	31.1 ± 3.4 (27.4–35.2)	19.9 ± 1.2 (18–22)	26.3 ± 1.9 (24–29)
Rectum length (RL)	22	23.8 ± 1.1 (22–25)	–	–	–	–	–	11.8 ± 2.1 (8.5–15.5)	–
RL/ABD	0.9	1.0 ± 0.1 (0.9–1.0)	–	–	–	–	–	0.6 ± 0.1 (0.4–0.8)	–
Tail length (TL)	165	154 ± 12.7 (133–166)	107 ± 4.3 (98–112)	117–143	77–92	159 ± 11.9 (125–180)	66 ± 7.4 (56–79)	111 ± 8.0 (101–127)	79.6 ± 5.3 (72–90)
Anus to phasmid	54	55 ± 6.2 (48–63)	55 ± 4.4 (49–60)	–	–	–	–	–	27.5 (n = 1)
Phasmid (% TL)	33	38 ± 6.7 (33–47)	51.8 ± 2.2 (50–55)	–	–	–	–	–	34 (n = 1)
Testis length	–	–	716 ± 50 (633–769)	–	–	–	–	–	542 ± 40 (453–588)
Testis flexure length	–	–	128 ± 23 (90–157)	–	–	–	171 ± 25.5 (133–199)	–	93.1 ± 15.3 (70–113)
Spicule length	–	–	32.0 ± 1.5 (31–35)	–	27–29	–	31.6 ± 1.8 (28.6–35.0)	–	27.4 ± 0.9 (26.5–29.0)
Gubernaculum length	–	–	28.5 ± 0.5 (28–29)	–	19–23	–	32.0 ± 2.6 (25.4–36.6)	–	25.4 ± 1.0 (24.0–26.5)

Measurements in µm and in the form mean ± standard deviation and (range) or only range

^a^Measured on drawings in the original publication; ^b^ Calculated from data in the original publication

*Abbreviations*: L, body length; a, body length divided by greatest body diameter; b, body length divided by the distance from anterior end to pharynx base; c, body length divided by tail length, c′, tail length divided by body diameter at level of anal or cloacal opening; V, position of the vulva from anterior end expressed as a percentage of total body length; T, length of male reproductive system expressed as a percentage of total body length

*Female*. Reproductive system didelphic, amphidelphic, ovaries reflexed, ovary flexures reaching vulval region. Oviducts filled with sperm. Gonads filled with oöcytes and hatched juveniles. Vulva a transverse slit, vulval lips not protruding, with epiptygmata; vagina *c*.1/4–1/3 of VBD. Tail conoid, elongate, tapering to a finely pointed terminus. Rectum short, less than one time ABD long. Phasmids are not discernible in our specimens.

*Male*. Similar to female in most respects, except for the sexual characters. Reproductive system monorchic, testis reflexed dorsad anteriorly. Spicules paired with weakly arcuate shaft and manubria bent sideways. Gubernaculum with robust dorsal apophysis. Pads on posterior lip of cloaca indistinct. Genital papillae distributed as follows: a single midventral large pad-like precloacal papilla 42–66 anterior to cloaca; 2 subventral precloacal pairs; 1 subventral adcloacal pair; 1 lateral pair near cloaca; and 1 subventral and 1 subdorsal caudal pair at midtail; other papillae indistinct. Phasmids are not discernible in our specimens. Tail differently shaped than in female, strongly curved ventrad, conoid with a 36–48 long mucro ending in a pointed terminus.

### Remarks

As in the Großform, the present material of the Kleinform of *A. appendiculatum* agrees well with the description by Mengert (1953) (see Table 5). Likewise, our study of the Californian specimens also largely corroborates the results obtained by Nermuť et al. (2015) with the Czech material. There are however some notable differences: the Czech specimens are generally of bigger size than the Californian specimens (body length 889–1,454 *vs* 781–905 µm for females; 848–1,010 *vs* 651–806 µm for males), and have longer spicules (28–35 *vs* 26–29 µm) and gubernaculum (25–37 *vs* 24–27 µm).

***Alloionema similis*** **n. sp.**

*Type-host*: *Arion hortensis* agg. (isolate ITD176).

*Other hosts*: *Arion hortensis* agg. (ITD175, ITD219 and ITD220), *Deroceras reticulatum* (ITD041, ITD216, ITD225 and ITD226) and *Lehmannia valentiana* (ITD197, ITD294 and ITD295).

*Type-locality*: Potted plant in a garden center in San Mateo, California, on 27.i.2013 (GPS coordinates: 37°34′18.62″N, 122°18′56.62″W).

*Type-material*: Holotype female, four paratype females and six paratype males on slide # SMNH-Type-8790 deposited in the invertebrate type collection of the Department of Zoology, Swedish Museum of Natural History, Stockholm, Sweden.

### Description of Kleinform cultured on agar (Figs. 1E–F, 3–5)

*General* [Based on 11 specimens; see measurements in Table 5.] Body 1.2–1.3 mm long in females and 0.9–1.0 mm long in males. When killed by heat, females almost straight and males slightly arcuate ventrad, more strongly arcuate in the posterior end. Cuticle finely annulated, annules less prominent in LM, *c.*1 wide. Lateral field not seen in LM or SEM. Anterior end gradually tapering. Lip region rounded, continuous with body contour. Six rounded lips grouped in 3 pairs, 1 dorsal and 2 ventrolateral, carrying 6 inner labial, 6 outer labial and 4 cephalic papilliform sensilla and 2 small oval amphids. Stoma somewhat longer than the lip region diameter. Cheilostom broad, with bacilliform rhabdia; gymnostom short; stegostom funnel-shaped, with strongly sclerotised lining and prominent denticles in its dorsal sector. Pharynx muscular; corpus cylindrical, 2.1–2.4 times as long as isthmus, widening posteriorly to a non-valvular metacorpus; isthmus narrower, demarcated by a break in muscular tissue; basal bulb oval, with strongly developed valves. Nerve-ring surrounding isthmus. Excretory pore opening at isthmus-bulb junction or at terminal bulb. Deirids not observed.

**Fig. 3:**
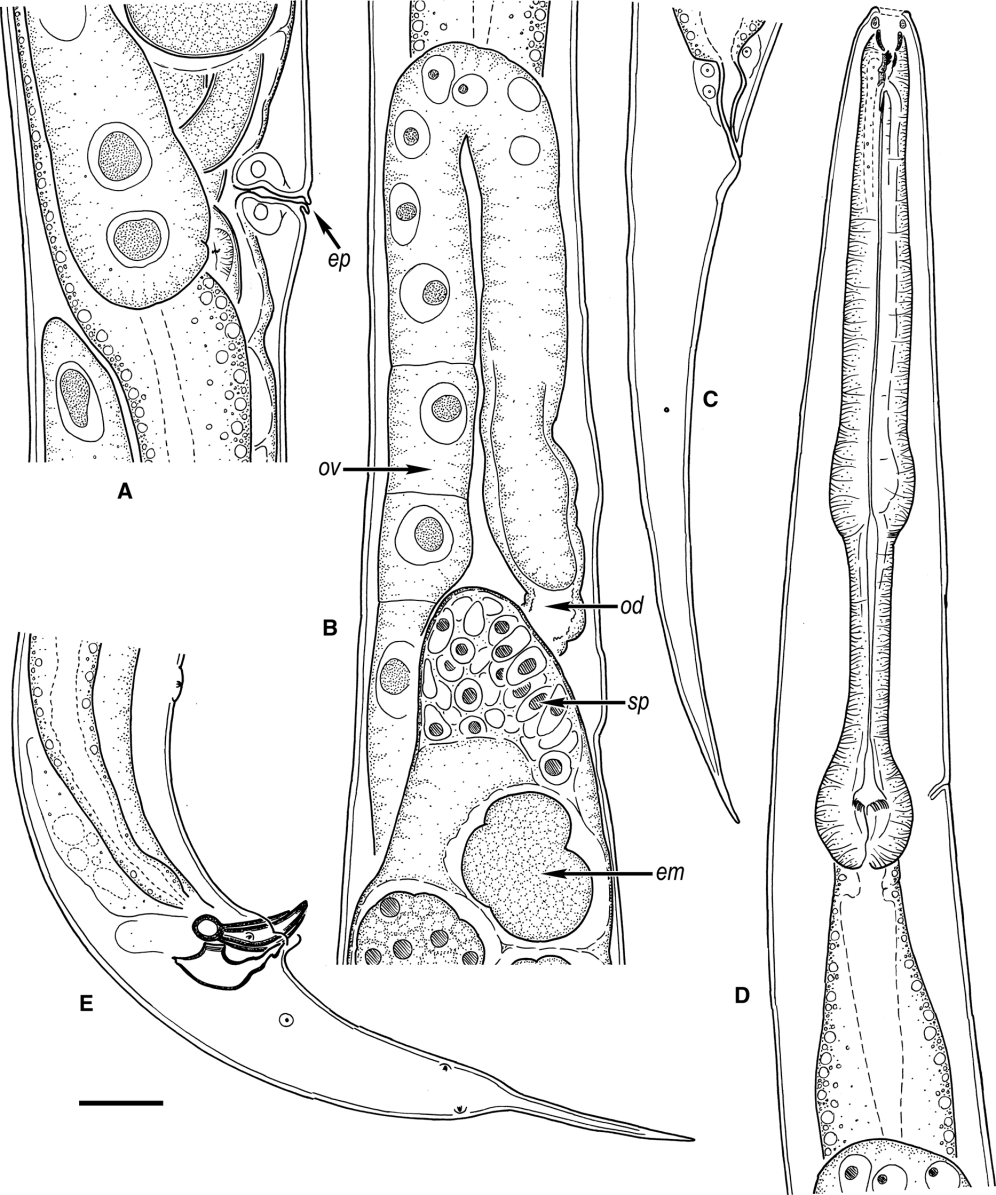
Line drawings of *Alloionema similis* n. sp. Kleinform generation. A, Vulval region showing epiptygmata (ep); B, Anterior ovary showing oöcytes of germinative zone (ov), oviduct (od), spermatheca (sp), and developing embryo (em); C, Female, tail; D, Female, pharyngeal region; E, Male, tail. *Scale-bar*: 20 µm

**Fig. 4:**
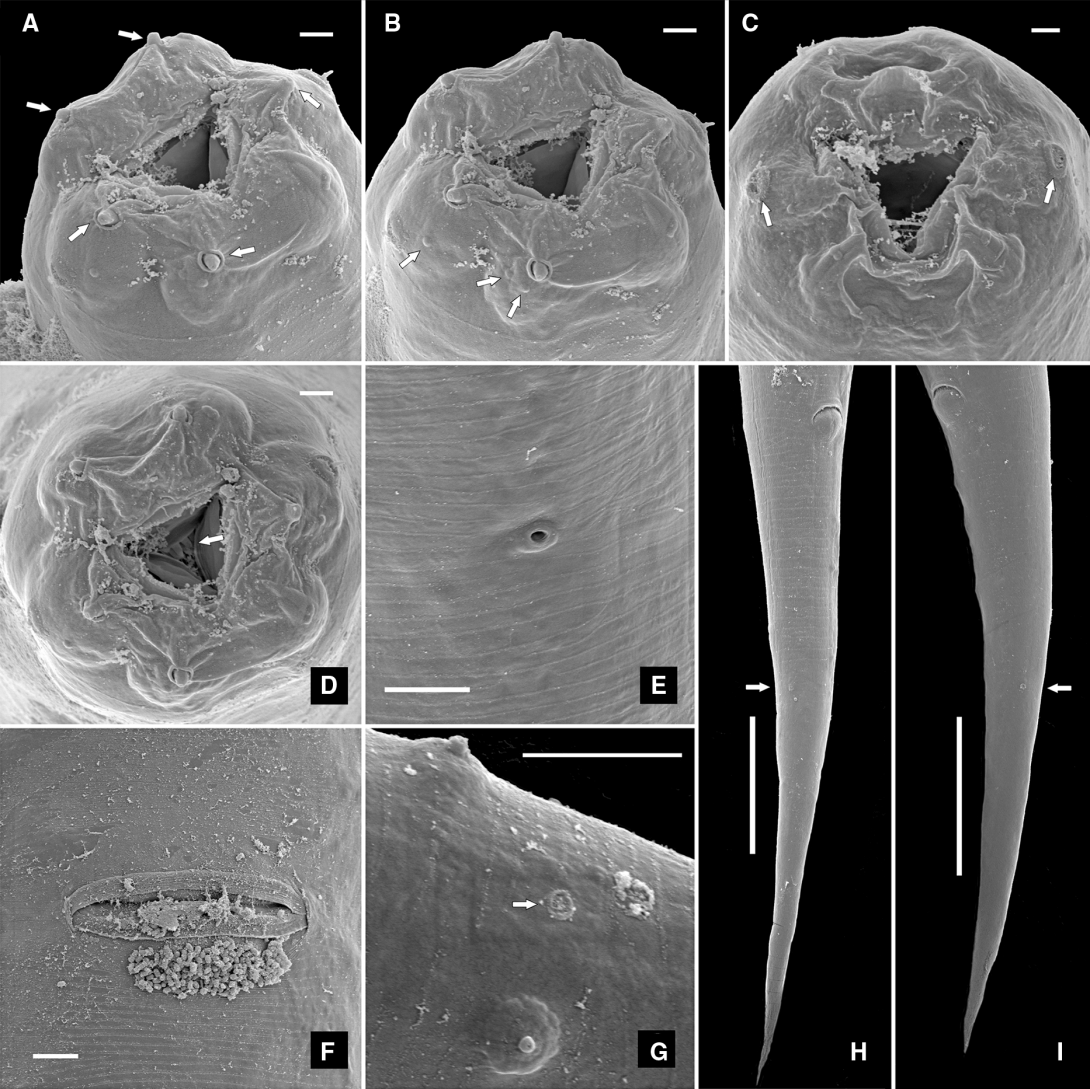
Scanning electron microscopy photomicrographs of the female and male Kleinform generation of *Alloionema similis* n. sp. A, Male, lip region (*arrows* point at inner labial sensilla); B, Male, lip region (*arrows* point at outer labial and cephalic sensilla); C, Female, lip region (*arrows* point at amphids); D, Male, lip region (*arrow* points at stegostomatal denticles); E, Excretory pore; F, Vulva; G, Phasmid (*arrow*) on male tail; H–I, Female, tail (*arrow* points at phasmid). *Scale-bars*: A–G, 5 µm; H–I, 25 µm

**Fig. 5:**
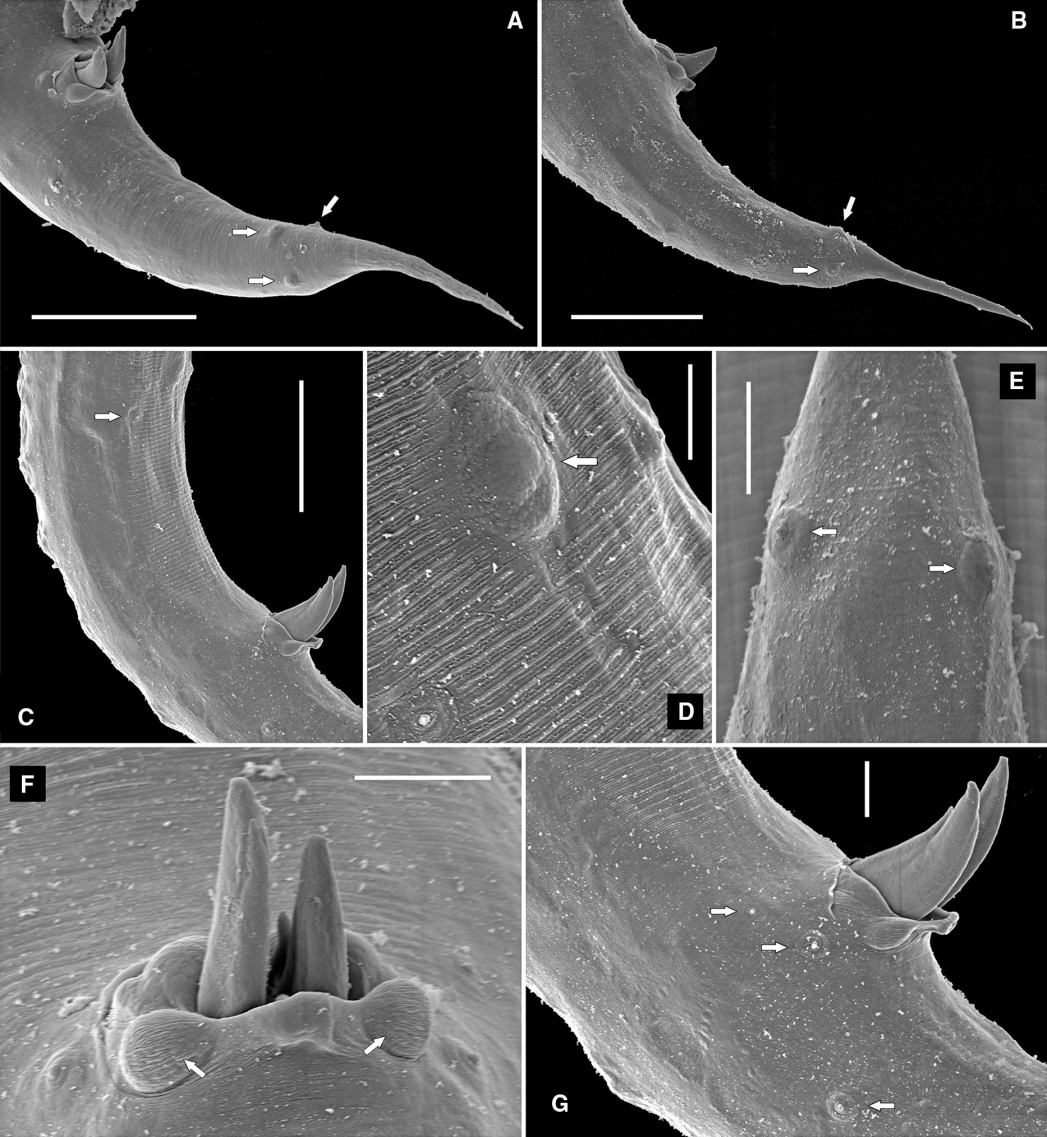
Scanning electron microscopy photomicrographs of the male Kleinform generation of *Alloionema similis* n. sp. A, B, Tail (*arrows* point at subventral and subdorsal caudal papillae); C, Cloacal region (*arrow* points at anteriormost precloacal subventral papilla); D, Single pad-like midventral papilla (*arrow*); E, Subdorsal caudal papillae (*arrows*); F, Ventral view of cloaca showing extruding spicules and two sublateral pads (*arrows*) on the posterior cloacal lip; G, Lateral view of cloaca showing extruding spicules, precloacal subventral papilla, adcloacal subventral papilla and anteriormost subventral caudal papilla (*arrows*). *Scale-bars*: A–C, 25 µm; D–G, 5 µm

*Female.* Reproductive system didelphic, amphidelphic, ovaries reflexed, posterior ovary flexure reaching almost to vulva. Oviducts filled with sperm. Gonads filled with oöcytes and hatched juveniles. Vulva a transverse slit, vulval lips not protruding, with epiptygmata; vagina *c*.1/3 of VBD. Tail conoid, elongate, tapering to a finely pointed terminus. Rectum short, about one time ABD long. Phasmids at one-third to half of tail length.

*Male*. Similar to female in most respects, except for the sexual characters. Reproductive system monorchic, testis reflexed dorsad anteriorly. Spicules paired, with weakly arcuate fusiform shaft and manubria bent sideways. Gubernaculum with robust dorsal apophysis. Two sublateral pads on the posterior lip of cloaca. Genital papillae distributed as follows: a single midventral large pad-like precloacal papilla 58–74 anterior to cloaca; 2 subventral precloacal pairs (at 54–66 and 15–22 anterior to cloaca, respectively); 1 subventral adcloacal pair; 1 lateral pair short distance posterior to cloaca; and 1 subventral and 1 subdorsal caudal pair at midtail. Phasmids at about half of tail length, between subventral and subdorsal caudal papillae. Tail differently shaped than in female, strongly curved ventrad, conoid with a 33–45 long mucro ending in a pointed terminus.

### Remarks

**Fig. 6:**
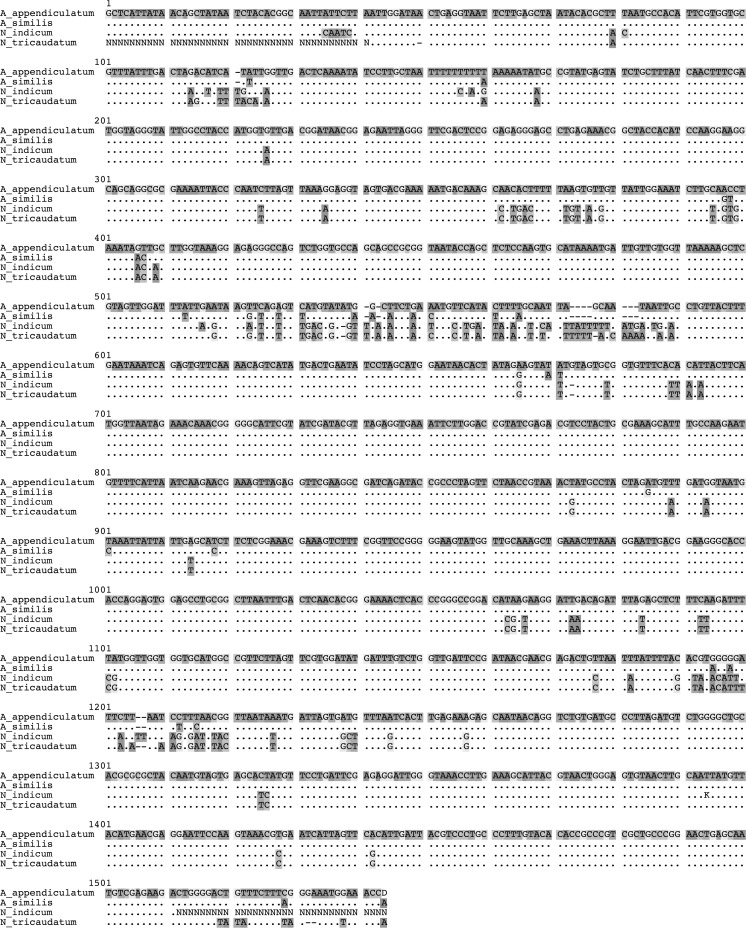
Secondary structure-based multiple sequence alignment of 18S rRNA of four species of the family Alloionematidae (consensus sequences of *Alloionema appendiculatum* and *A. similis* n. sp.), showing differences between species; *dots* indicate nucleotides identical to those in the top sequence, *dashes* indicate alignment gaps (indels)

**Fig. 7:**
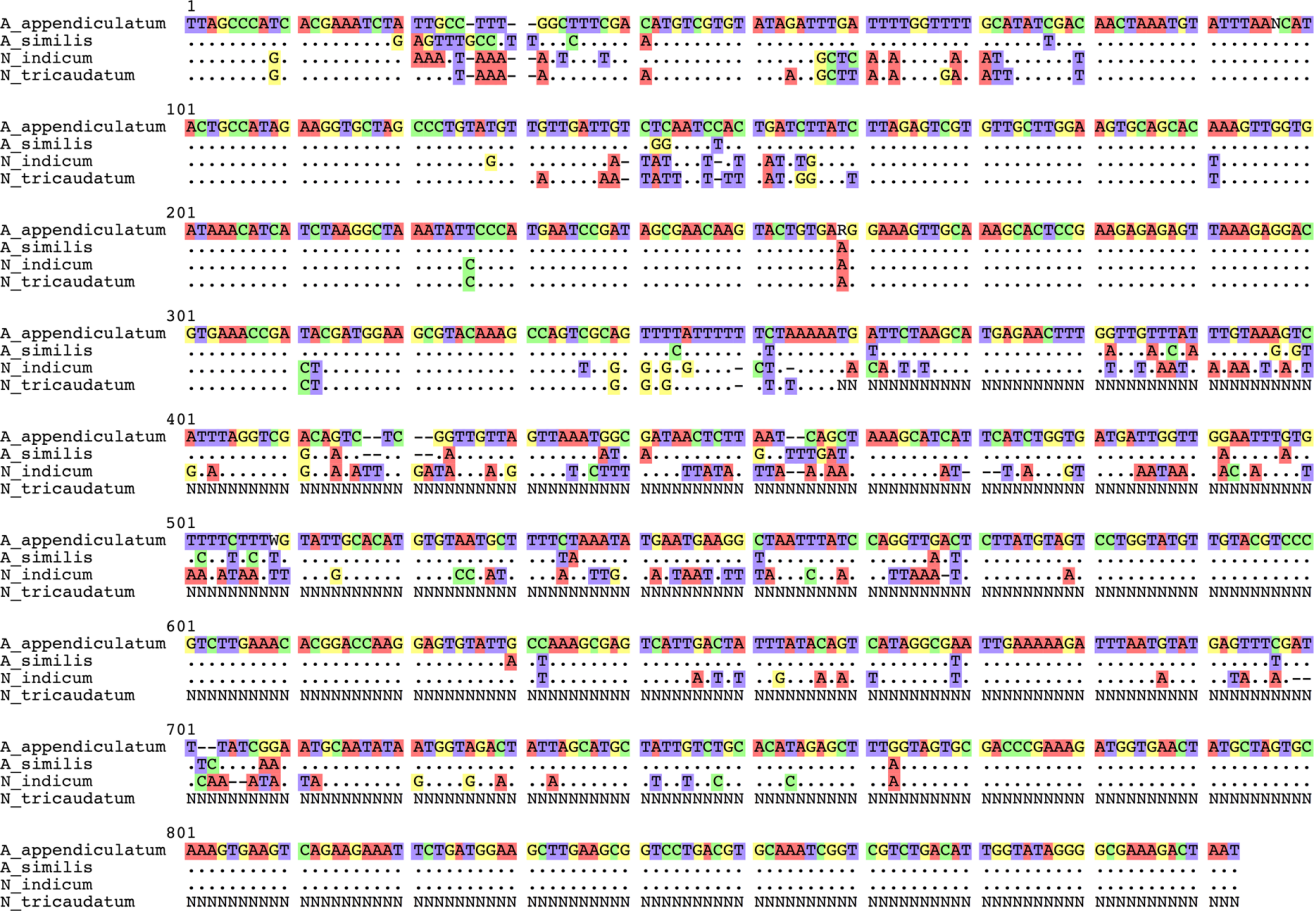
Secondary structure-based multiple sequence alignment of partial 28S rRNA of four species of the family Alloionematidae (consensus sequences of *Alloionema appendiculatum* and *A. similis* n. sp.), showing differences between species; *dots* indicate nucleotides identical to those in the top sequence, *dashes* indicate alignment gaps (indels)

**Fig. 8:**
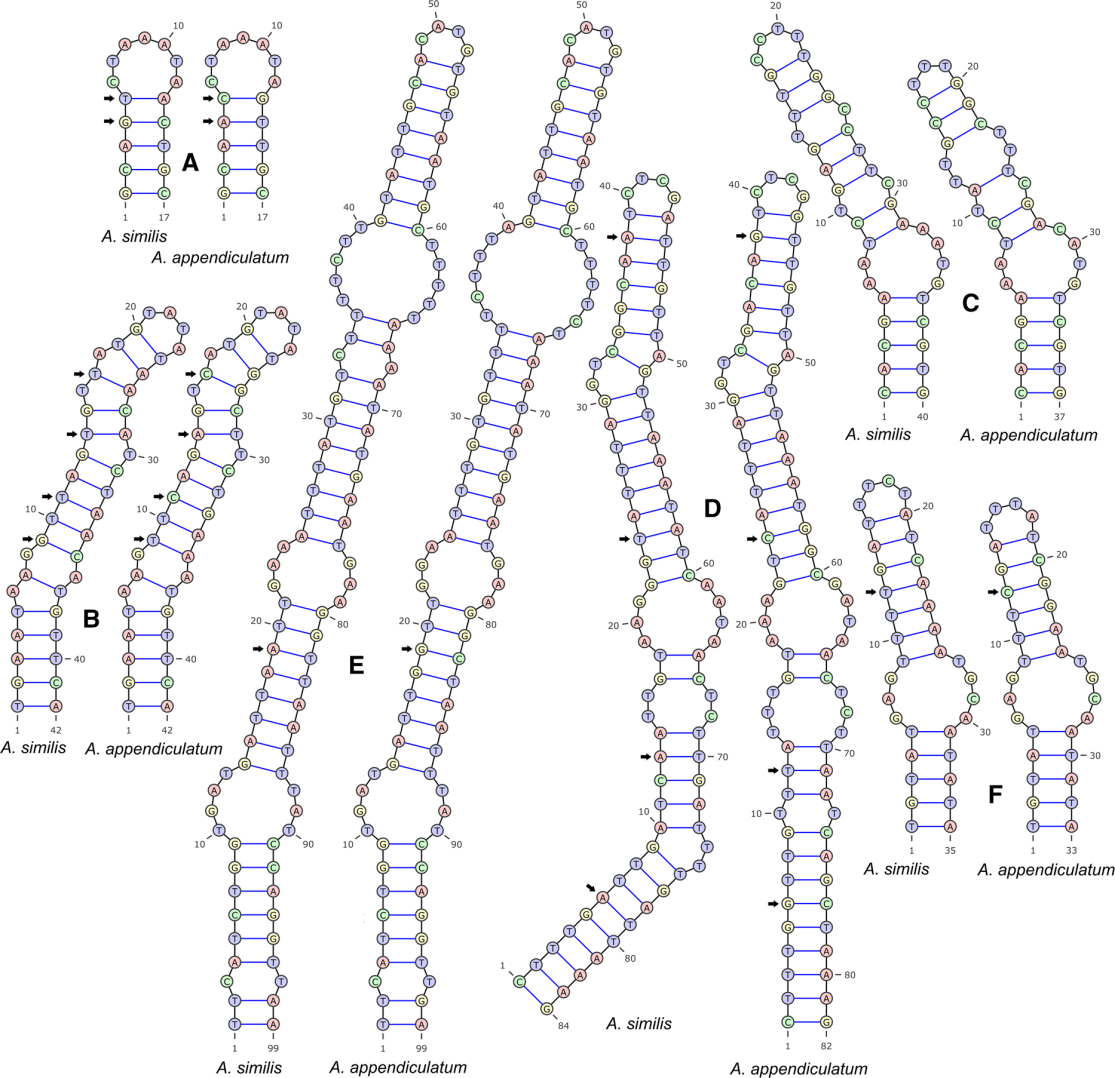
Structural differences in 18S rRNA (A, B) and partial 28S rRNA (C–F) helices between *Alloionema appendiculatum* and *A. similis* n. sp.; helices numbered according to Wuyts et al. (2001, 2002) and Chilton et al. (2003). A, helix 18; B, helix 23e/1-23e/2; C, helix b13_1; D, helix c2_b; E, helix c2_c; F, helix d5. Compensatory substitutions marked with arrows

**Fig. 9:**
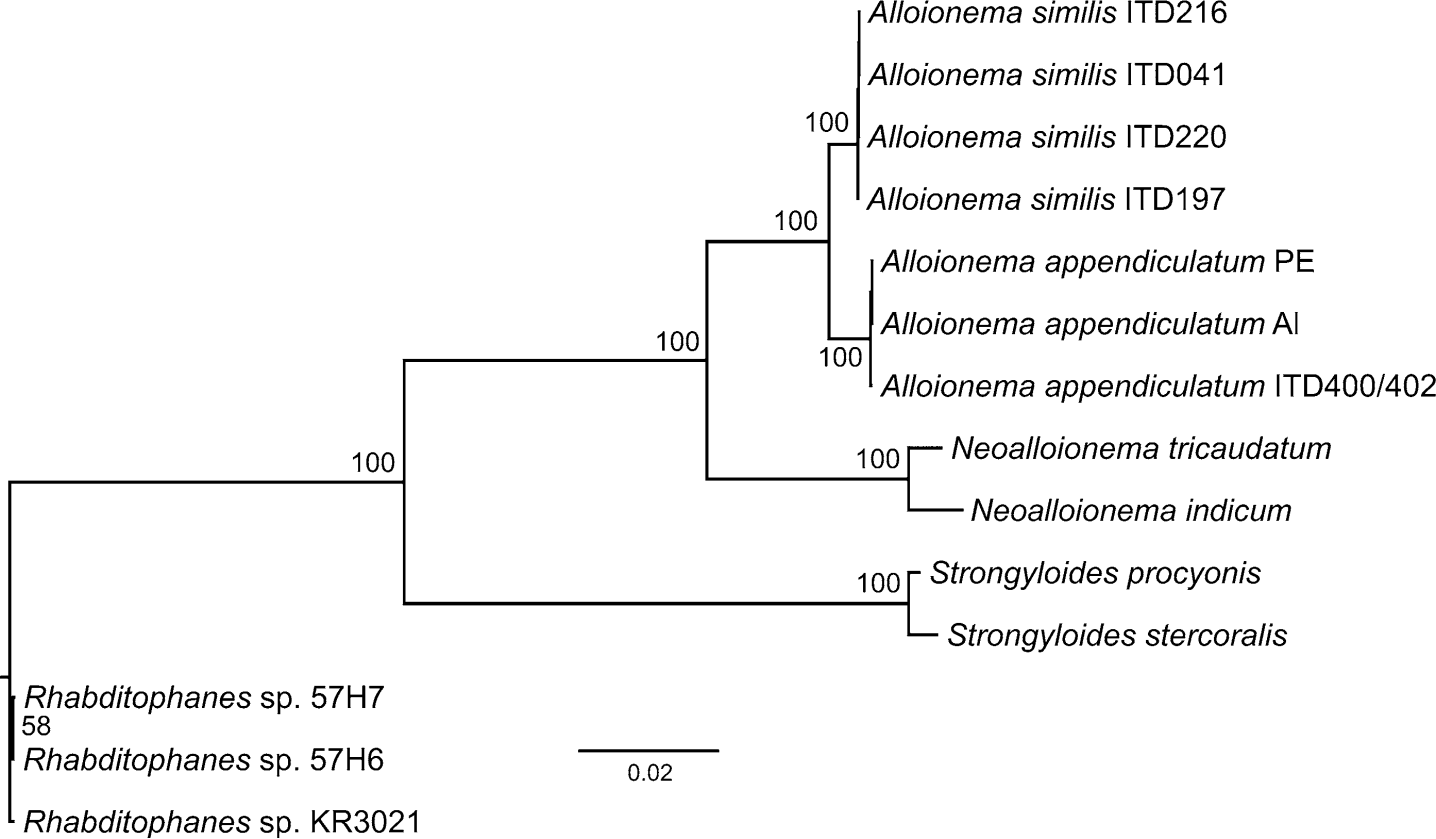
Majority-rule consensus tree of the Bayesian phylogenetic analysis of concatenated alignment of 18S rRNA and D1-D2-D3 domains of 28S rRNA, rooted using *Rhabditophanes* sp. KR3021, branch lengths represent the mean posterior estimates of the expected number of substitutions per site

The new species is morphologically very similar to *Alloionema appendiculatum*, hence it is given the name *Alloionema similis* n. sp. Morphologically, it agrees well with the description by Mengert (1953) (see Table 5), except for the size of the Kleinform which are generally bigger in this study (body length 1,189–1,309 *vs* 922–1,073 µm for females; 910–1,029 *vs* 561–926 µm for males). Similar size differences were obtained by Nermuť et al. (2015) and a reason for these variations could be the food source or culture media used. Another difference is the number of male genital papillae, which were recorded as being five by Mengert (1953), but were revealed by SEM to be six (Nermuť et al., 2015; this paper). The new species is morphologically very similar to the Czech specimens of *A. appendiculatum* described by Nermuť et al. (2015), but there are some differences: (i) more posterior position of the excretory pore in the Kleinform females (169–188 *vs* 117–164 µm from anterior end); (ii) smaller anal body diameter in Kleinform females (24–26 *vs* 27–39 µm); and (iii) longer tail in Kleinform males (98–112 *vs* 56–79 µm; c = 9.2–9.9 *vs* 11.4–17.8; c′ = 3.0–3.4 *vs* 3.9–6.9). There are some problems with the latter comparison since the tail length 56–79 µm and anal body diameter 27.4–35.2 µm will give a c′ of about 2 and not 3.9-6.9, thus a mistake is possibly made in Table 1 of Nermuť et al. (2015). There are, however, substantial differences in both 18S and 28S rDNA sequences between *Alloionema similis* n. sp. and *A. appendiculatum* (Figs. 6–9), which will be discussed in the next section.

Our material sheds no light on the validity or status of *A. appendiculatum* var. *dubia* (Chitwood & McIntosh, 1934), which was described only from Großform. These adults were smaller in size and had a noticeably longer basal bulb than any of the material listed in our Table 1. All prior descriptions of *A. appendiculatum* (*sensu stricto*), as well as our own measurements, indicate that the basal bulb length is comparable in Kleinform and Großform. In view of the smaller body length of *A. appendiculatum* var. *dubia*, particularly in males, we are confident that *Alloionema similis* n. sp. does not represent the same organism as the nematode described by Chitwood & McIntosh (1934). It is also worth noting that these authors illustrated the lip region and subcephalic region of *A. appendiculatum* var. *dubia* as being cylindrical rather than clearly tapering, which is a condition not seen in any of our material nor shown in any of the illustrations by Nermuť et al. (2015). For the time being we therefore consider it best to neither elevate *A. appendiculatum* var. *dubia* to a separate species, nor to treat as a match with *A. appendiculatum* (*sensu stricto*). The resolution of its status must await new collections from *Succinea* snails obtained near the location reported by Chitwood & McIntosh (a swamp near Piscataway in Maryland).

## Discussion

### Interspecific variability of ribosomal RNA gene sequences

The four-taxa secondary-structure based multiple sequence alignments of 18S and 28S rRNA genes of all current members of the family Alloionematidae contained 1,544 and 893 positions respectively. There were 27 (12 within 18S and 15 within 28S rDNA) unambiguous autapomorphies for *Alloionema similis* n. sp.; 26 (20 within 18S and 6 within 28S rDNA) for *A. appendiculatum*; 31 (21 within 18S and 10 within 28S rDNA) for *Neoalloionema indicum*; and 24 (14 within 18S and 10 within 28S rDNA) for *N. tricaudatum* (Figs. 6–7). Not all apomorphies can be accounted for within the 28S rRNA gene due to the fact that large part of the gene was not sequenced for *N. tricaudatum.* Molecular differences between *Alloionema appendiculatum* and *A. similis* n. sp. are not limited to random mutations, but include a number of compensatory substitutions in the hairpins 18, 23e/1-23e/2, b13_1, c2_b, c2_c and d5 of the secondary structure of both 18S and 28S rRNA genes (Fig. 8).

### Phylogenetic analysis

The combination of characters: stoma short with sclerotised anterior part and non-sclerotised funnel-shaped posterior part, median bulb without valves, basal bulb with valves, female reproductive system didelphic and amphidelphic with reflexed ovaries, and male tail without bursa, makes the systematic position of *Alloionema* somewhat uncertain. For *Alloionema* and *Rhabditophanes* Fuchs, 1930, Chitwood & McIntosh (1934) proposed a new subfamily in the family Diplogastridae Micoletzky, 1922, the Alloionematinae Chitwood & McIntosh, 1934, differing from Diplogastrinae by the presence of a basal bulb with valves. Goodey (1963) placed Alloionematinae in the family Rhabditidae Örley, 1880 while Andrássy (1976) raised it to superfamily and family level (Alloionematoidea Chitwood & McIntosh, 1934 and Alloionematidae Chitwood & McIntosh, 1934) in Rhabditina. Based on molecular characters, De Ley & Blaxter (2004) placed Alloionematidae in the superfamily Strongyloidoidea Chitwood & McIntosh, 1934 in the infraorder Panagrolaimomorpha De Ley & Blaxter, 2004. In a study on the molecular phylogeny of slug-parasitic nematodes, based on 18S rRNA gene sequences, *A. appendiculatum* clustered in a clade with species of *Strongyloides* Grassi, 1879 and *Rhabditophanes* Fuchs, 1930 (see Ross et al., 2010). In the study by Nermuť et al. (2015), the molecular evidence from several ribosomal genes also generated a strongly supported clade including *A. appendiculatum* and species of *Strongyloides*, *Parastrongyloides* Morgan, 1928 and *Rhabditophanes*. In our analysis (Fig. 9) both *A. appendiculatum* and *A. similis* were placed as sister taxa in a strongly supported clade. *Neoalloionema tricaudatum* Ivanova, Pham Van Luc & Spiridonov, 2016 and *N. indicum* Nermuť, Půža & Mráček, 2016. formed a distinct strongly supported clade, in agreement with a recent study published by Nermuť et al. (2016).

## References

[CR1] Andrássy, I. (1976). *Evolution as a basis for the systematization of nematodes*. London, UK: Pitman Publishing, 288 pp.

[CR2] Blaxter ML, De Ley P, Garey JR, Liu LX, Scheldeman P (1998). A molecular evolutionary framework for the phylum Nematoda. Nature.

[CR3] Chilton NB, Huby-Chilton F, Gasser RB (2003). First complete large subunit ribosomal RNA sequence and secondary structure for a parasitic nematode: phylogenetic and diagnostic implications. Molecular and Cellular Probes.

[CR4] Chitwood BG, McIntosh A (1934). A new variety of *Alloionema* (Nematoda: Diplogasteridae), with a note on the genus. Proceedings of the Helminthological Society of Washington.

[CR5] Claus C (1868). Beobachtungen ueber die Organisation und Fortplanzung der *Leptodera appendiculata*. Schriften der Gesellschaft zur Beförderung der gesammten Naturwissenschaften in Marburg.

[CR6] Darty K, Denise A, Ponty Y (2009). VARNA: Interactive drawing and editing of the RNA secondary structure. Bioinformatics.

[CR7] De Ley P, Blaxter ML (2004). A new system for Nematoda: combining morphological characters with molecular trees, and translating clades into ranks and taxa. Nematology Monographs and Perspectives.

[CR8] Goodey, T. (1963). *Soil and freshwater nematodes*. 2nd Ed., rewritten by J. B. Goodey. London, UK: Methuen & Co Ltd, 544 pp.

[CR9] Gouy M, Guindon S, Gascuel O (2010). SeaView version 4: a multiplatform graphical user interface for sequence alignment and phylogenetic tree building. Molecular Biology and Evolution.

[CR10] Gowri-Shankar, V., & Jow, H. (2006). *PHASE: a software package for Phylogenetics and Sequence Evolution*. Manchester, UK: University of Manchester, 60 pp.

[CR11] Higgs PG (2000). RNA secondary structure: physical and computational aspects. Quartly Reviews of Biophysics.

[CR12] Holovachov O, Camp L, Nadler SA (2015). Sensitivity of ribosomal RNA character sampling in the phylogeny of Rhabditida. Journal of Nematology.

[CR13] Ivanova E, Van Luc Pham, Spiridonov S (2016). *Neoalloionema tricaudatum* gen. n., sp. n. (Nematoda: Alloionematidae) associated with a cyclophorid snail in Cuc Phuong Natural Park, Vietnam. Nematology.

[CR14] Laznik Z, Ross JL, Tóth T, Lakatos T, Vidrih M, Trdan S (2009). First record of the nematode *Alloionema appendiculatum* Schneider (Rhabditida: Alloionematidae) in Arionidae slugs in Slovenia. Russian Journal of Nematology.

[CR15] Laznik Z, Ross JL, Trdan S (2010). Massive occurrence and identification of the nematode *Alloionema appendiculatum* Schneider (Rhabditida: Alloionematidae) found in Arionidae slugs in Slovenia. Acta Agriculturae Slovenica.

[CR16] Mc Donnell, R. J., Paine, T. D., & Gormally, M. J. (2009). *Slugs: A Guide to the Invasive and Native Fauna of California*. University of California Division of Agriculture and Natural Resources, Davis, California, 21 pp.

[CR17] Mengert H (1953). Nematoden und Schnecken. Zeitschrift für Morphologie und Ökologie der Tiere.

[CR18] Nermuť J, Půža V, Mráček Z (2015). Re-description of the slug-parasitic nematode *Alloionema appendiculatum* Schneider, 1859 (Rhabditida: Alloionematidae). Nematology.

[CR19] Nermuť, J., Půža, V., & Mráček, Z. (2016). *Neoalloionema indicum* n. sp. (Nematoda: Alloionematidae), a new alloionematid from India. *Nematology, 18*, in press. 10.11646/zootaxa.4184.3.5 27988776

[CR20] Ross JL, Ivanova ES, Spiridonov SE, Waeyenberge L, Moens M, Nicol GW, Wilson MJ (2010). Molecular phylogeny of slug-parasitic nematodes inferred from 18S rRNA gene sequences. Molecular Phylogenetics and Evolution.

[CR21] Schneider A (1859). Ueber eine Nematodenlarve und gewisse Verschiedenheiten in den Geschlechtsorganen der Nematoden. Zeitschrift für wissenschaftliche Zoologie.

[CR22] Schneider, A. (1866). *Monografie der Nematoden*. Berlin, Germany: Verlag von Georg Reimer, 357 pp.

[CR23] Seibel PN, Müller T, Dandekar T, Schultz J, Wolf M (2006). 4SALE – A tool for synchronous RNA sequence and secondary structure alignment and editing. BMC Bioinformatics.

[CR24] Tandingan De Ley I, De Ley P, Vierstaete A, Karssen G, Moens M, Vanfleteren J (2002). Phylogenetic analyses of *Meloidogyne* small subunit rDNA. Journal of Nematology.

[CR25] Tandingan De Ley I, McDonnell RD, Lopez S, Paine TD, De Ley P (2014). *Phasmarhabditis hermaphrodita* (Nematoda: Rhabditidae), a potential biocontrol agent isolated for the first time from invasive slugs in North America. Nematology.

[CR26] Tandingan De Ley I, Mundo-Ocampo M, Yoder M, De Ley P (2007). Nematodes from vernal pools in the Santa Rosa Plateau Ecological Reserve, California I. *Hirschmanniella santarosae* sp. n. (Nematoda: Pratylenchidae), a cryptic sibling species of *H. pomponensis* Abdel-Rahman & Maggenti, 1987. Nematology.

[CR27] Tavaré S (1986). Some probabilistic and statistical problems on the analysis of DNA sequences. Lectures on Mathematics in the Life Sciences.

[CR28] Wuyts J, De Rijk P, Van de Peer Y, Winkelmans T, De Wachter R (2001). The European Large Subunit Ribosomal RNA database. Nucleic Acids Research.

[CR29] Wuyts J, Van de Peer Y, Winkelmans T, De Wachter R (2002). The European database on small subunit ribosomal RNA. Nucleic Acids Research.

